# MicroRNA as a Diagnostic Tool, Therapeutic Target and Potential Biomarker in Cutaneous Malignant Melanoma Detection—Narrative Review

**DOI:** 10.3390/ijms24065386

**Published:** 2023-03-11

**Authors:** Agata Poniewierska-Baran, Łukasz Zadroga, Edo Danilyan, Paulina Małkowska, Paulina Niedźwiedzka-Rystwej, Andrzej Pawlik

**Affiliations:** 1 Institute of Biology, University of Szczecin, Felczaka 3c, 71-412 Szczecin, Poland; 2 Department of Physiology, Pomeranian Medical University in Szczecin, Powstancow Wielkopolskich 72, 70-111 Szczecin, Poland; 3 Biology Department, Faculty of Science and Data Analytics, Institut Teknologi Sepuluh Nopember, Surabaya 60111, Indonesia; 4 Doctoral School of the University of Szczecin, 71-412 Szczecin, Poland

**Keywords:** miRNA, melanoma, biomarker, skin cancer, metastasis, clinical trials

## Abstract

Melanoma is the most serious type of skin cancer, causing a large majority of deaths but accounting for only ~1% of all skin cancer cases. The worldwide incidence of malignant melanoma is increasing, causing a serious socio-economic problem. Melanoma is diagnosed mainly in young and middle-aged people, which distinguishes it from other solid tumors detected mainly in mature people. The early detection of cutaneous malignant melanoma (CMM) remains a priority and it is a key factor limiting mortality. Doctors and scientists around the world want to improve the quality of diagnosis and treatment, and are constantly looking for new, promising opportunities, including the use of microRNAs (miRNAs), to fight melanoma cancer. This article reviews miRNA as a potential biomarker and diagnostics tool as a therapeutic drugs in CMM treatment. We also present a review of the current clinical trials being carried out worldwide, in which miRNAs are a target for melanoma treatment.

## 1. Introduction

Cutaneous malignant melanoma (CMM) is one of the most aggressive and progressive types of skin cancer. Two growth phases of melanoma have been identified—radial and vertical. In the radial growth phase, melanoma appears as an irregular plaque, whereas in the vertical growth phase, the lesion develops vertically, forming a tumor [1]. So far, no specific and sensitive clinical biomarkers for the diagnosis or prognosis of melanoma patients have been established. Although there are some studies that describe the correlation of various molecules with the occurrence of melanoma, it has not been enough. These factors/molecules must follow strict criteria to be called biomarkers. First of all, a biomarker by definition needs to be specific to the particular pathology and sensitive enough to avoid false positive or negative diagnosis. It is best if the melanoma’s presence is detected before the clinical symptoms present or in the early stages of cancer development, so its concentration should depend on disease progression or response to treatment. Of course, it should be available, easily quantified in the body fluid, and last but not least, it should be translatable from research to the clinic. To confirm whether a given molecule can be considered a biomarker, a series of calculations must be performed, i.e., correlation between the presence or level of the potential biomarker and the prognostic indicators of melanoma, such as distant metastasis free survival (DMFS), overall survival (OS), disease-free survival (DFS), relapse-free survival (RFS) and melanoma-specific survival (MSS). It is important to note that according to new generation sequencing, being a useful tool for diagnostic purposes, multiple or reoccurring mutations’ characteristic for melanoma have been identified, most of which are enriched in C>T mutations representing UV-driven mutagenesis [2]. Moreover, genetic alterations, such as oncogenic BRAF or NRAS, leading to the mitogen-activated protein kinase pathway activation, are the most common early events in melanomas of the skin. The difficulty of differentiation is hidden in this aspect, as these are also present in melanocytic nevi [3]. For a prompt comparison of melanomas against melanocytic nevi, molecular patterns of these two separate diseases have been now established [4]. Differentiation with mass spectrometry-based proteomics reported a distinctive panel of biomarkers for amelanotic aggressive melanoma [5]. It turns out that a different molecular pattern is employed in the invasive melanoma in comparison with less aggressive forms of disease. Moreover, an in situ characterization has been performed, confirming the heterogeneity of eIF4F phenoclusters in multiple human melanoma cell lines, and it was found that specific eIF4F localization patterns may be related to chemoresistance in cancer treatment [6]. An additional tool for differential diagnosis may also be PRAME immunohistochemistry [7].

## 2. Biological Characteristics of MicroRNA

MicroRNAs, also known as miRNAs, are small (18–24 nt), single-stranded, non-coding RNAs that can regulate the translation (the process of protein biosynthesis) of mRNA, which is of great importance in the regulation and control of a cell apoptosis, stress responses, survival and differentiation [8]. MicroRNAs belong to the most numerous group of molecules that affect the regulation of genes in animals, which makes it obvious that their role in the development of many diseases, including cancer, is significant [9]. The deregulation of miRNA has been described for almost every aspect of melanoma formation and progression, tumor growth, angiogenesis and metastasis, which we described previously [10]. Metastasis of melanoma, or any type of cancer, is a multistage process in which the neoplastic cells leave the primary tumor, travel through the blood and/or lymphatic vessels, settle in distant organs and create secondary tumors. MicroRNA (miRNA) participate in several steps of these metastatic process [10].

We distinguish two types of microRNA biogenesis, described as canonical and non-canonical biogenesis. In the canonical biogenesis pathway, primary miRNAs (pri-miRNA) are transcribed from genes by RNA polymerase III. Processing them into precursor miRNA (pre-miRNAs) is conducted by the Microprocessor complex of two multiprotein units, the first being a large multiprotein unit that includes double-stranded RNA (dsRNA) binding proteins, ribonucleoproteins and Ewing’s sarcoma protein family, and the other one being a small multiprotein unit that includes the RNase III enzyme (Drosha) and the RNA-binding protein DiGeorge Syndrome Critical Region 8 (DGCR8) [11,12]. Pre-miRNAs are transferred from the nucleus to the cytoplasm by a complex of exportin5 (XPO5) and RAS-related nuclear protein-guanosine-5′-triphosphate-ase (Ran-GTPase) [13]. In the cytoplasm, the terminal loop of pre-miRNA is removed by Dicer (the RNase III endonuclease). MicroRNAs, as short RNA duplexes (miRNA duplexes), are loaded into the RNA-induced silencing complex (RISC) [14]. This complex, when linked with miRNA (as miRISC), can inhibit multiple steps of protein synthesis and affect the mRNA target stability [15]. Typically, the target site of the mRNA is in the 3′ untranslated region (3′UTR) and the complementary sequence on the miRNA (called the seed sequence), and the non-canonical seed regions have been identified, such as in the 5′UTR sequence and the coding regions of mRNAs [16]. In the non-canonical miRNA biogenesis pathway, functionally miRNAs result from a different combination of the same proteins of the canonical pathway. We distinguish two main non-canonical miRNA biogenesis pathways: Drosha/DGCR8-independent (i.e., miRtron-derived pri-miRNAs) and Dicer-independent (i.e., pre-miR-451) [17], but there could also be small nucleolar RNAs (snoRNAs) and transfer RNAs (tRNAs) [18]. The role of non-canonical miRNA in biological functions and cancer development is poorly understood, but splicing factors, such as SRSF1 and SRSF2, are known to alter miRtron-derived miRNA levels. MicroRNAs mediate post-transcriptional silencing either through destabilization of the target mRNA, or repression of translation [19,20].

Due to the large amount of data indicating the involvement of miRNA molecules in the development of cancer, many studies have been conducted comparing the expression profiles of miRNAs (in various types of cancers) with the corresponding normal tissues [21,22,23], including melanocytes and melanoma cancer cells [24]. Although in our work we present many results of studies, showing altered miRNA expression in malignant melanoma, there is still a lack of studies comparing the wide range of miRNA molecules examined in normal human epidermal melanocytes (NHEM) vs. established melanoma cell lines. Mueller et al. [24] performed miRNA expression arrays comparing NHEMs with melanoma cell lines derived from melanoma primary tumors and metastases. They identify sets of miRNAs with altered expression during malignant transformation as well as in the progression of the disease and in metastasizing events. They identify a cohort of 49 miRNAs strongly upregulated during early progression, and found six miRNAs upregulated (at least three-fold) from melanocytes to primary melanoma, as well as from primary melanoma to metastatic melanoma cell lines miR-133a, miR-199b, miR-453, miR-520f, miR-521 and miR-551b, but also miR-190, whose expression was downregulated from melanocytes to primary melanoma, and from primary melanoma to metastatic melanoma cell lines [24]. Data derived from the miRNA microarray experiments were then verified using a quantitative real-time PCR (qRT-PCR) analysis, which showed that miR-17-5p, miR-222, miR-181a, miR-194, miR-22 and miR-373 expression in melanocytes and melanoma cell lines was in agreement with the data obtained from the miRNA microarray experiments. It is known that miRNAs are important players in cancer development and progression. Cancer cells have dysregulated miRNA expression profiles compared to their “normal” physiological counterparts. Studies have shown that miRNAs can act both as tumor suppressors but also as oncogenes depending on their target genes [25]. Upregulation of oncogenic miRNAs (oncomiRs) through the amplification and translocation of miRNA genes, and downregulation of oncosuppressor miRNAs (anti-oncomiRs) through deletion and other mutations, promoter methylation and abnormal processing, can contribute to melanoma initiation and progression [9,25]. A number of tumor suppressive miRNAs have been shown to play essential roles in melanoma cancer, such as miR-137, miR-101, miR-26a, miR-196a, miR-125, the miR-200 family, miR-205, miR-203 and miR-211 [26,27,28,29,30]. It was found that expression of the receptor tyrosine kinase c-KIT decreased with melanoma progression [31], and that miR-221/222 expression increased with tumor progression and was inversely correlated with c-KIT expression [32]. Looking at the current results, we can also now include oncogenes determining the development of melanoma such as miR-21, miR-155, miR-182 and miR-214 [33,34,35].

It is important to use new molecular approaches to find novel biomarkers to better prevent and diagnose melanoma. Studies have shown that miRNA in blood samples can distinguish between cancer-free and cancer-burdened patients, offering new hope for miRNA use as an early detection marker.

## 3. MicroRNAs as Blood-Based Melanoma Markers

MicroRNAs are exceedingly stable molecules in the bloodstream that are excreted from exosomes/microvesicles, and as a result they are protected against degradation by endogenous RNAses [36,37]. In melanoma patients, a study by Fogli et al. [38] found increased levels of miR-150-5p and miR-149-3p, and downregulated levels of miR-193a-3p, miR-15b-5p and miR-524-5p. As a viable method for melanoma diagnosis, scientists suggested the combination of miR-150-5p, miR-149-3p and miR-193a-3p, which had a 94.8% sensitivity and 83.9% specificity [38]. Patients with stage 0 melanoma had downregulated levels of miR-134-5p and miR-320a-3p as compared to healthy controls, according to an analysis of miRNA expression levels in plasma samples. Several miRNAs were expressed at lower levels in stage 0 melanoma patients compared to healthy controls, and at considerably lower levels in stage I/II patients. With 96% specificity and 90% sensitivity, measurements of miR-134-5p and miR-320a-3p levels have been proposed as diagnostic biomarkers [39]. Van Laar et al. [40] profiled the signature expression of 38 miRNAs obtained from the plasma of melanoma patients and healthy signatures that were differentially expressed in melanoma and normal plasma samples. Several molecular pathways involved in melanoma carcinogenesis and metastasis were modulated by genes in the signature panel [40,41,42,43,44,45]. In a more recent study by Van Laar et al. [46], the MEL-38 panel was able to identify melanomas from lower-risk skin lesions in formalin-fixed paraffin-embedded samples with 89% sensitivity and 94% specificity, suggesting its potential role as an accurate diagnostic biomarker for cutaneous melanoma [46].

Exosome-derived miRNAs (EV-miRNAs) are a novel area of interest in melanoma research, since they may serve as a diagnostic biomarker due to their presence in all bodily fluids and their small size [47]. By comparing microarray data from plasma samples of melanoma patients and healthy controls, Xiong et al. [48] were able to identify 55 dysregulated exosome-derived miRNAs. MiR-765, miR-362-3p, miR-550a-3p, miR-3907 and miR-500a-3p were among the miRNAs with the most elevated levels, as determined using the *p*-value, while miR-1238, miR-1228-3p, miR-10a-5p and miR-150-5p were the most negatively regulated miRNAs [48]. The results of the study conducted by Guo et al. [49] showed a decrease in the plasma levels of exosome miR-1180-3p. According to research, miR-1180-3p decreased the level of the ST3GAL4 gene in the body. The manipulation of miR-1180-3p expression allowed for the identification of the ST3GAL4 boosting effect on melanoma cell migration [49].

In order to distinguish melanoma brain metastases from other brain metastases and glioblastoma, the expression levels of numerous plasma miRNAs were measured and compared to those of control participants. A study found that the expression of 164 plasma miRNAs was significantly altered in melanoma patients. The largest levels of upregulated miRNAs were found for miR-223-3p, miR-92a-3p, miR-6803-3p, miR-26a-5p and miR-21-5p when compared to healthy donors. The authors identified a group of miRNAs, including miR-5694, miR-6796-3p, miR-6741-3p, miR-4664-3p, miR-4665-5p and miR-671-5p, which could identify the brain metastases of melanoma from the lung, breast cancer metastasis and glioblastoma [50]. Another set of 51 metastasis-related miRNAs was found in a study by Armand-Labit et al. [51] by measuring their frequency rate in the patient/control ratio. Out of 51 miRNAs, miR-103, miR-423-3p and miR-191 were shown to be the most stable, and their potential as diagnostic biomarkers was also examined. Even though the diagnostic performance of each individual miRNA was not established, the combination of miR-185 and miR-1246 was substantially associated with greater accuracy as biomarkers of metastatic cutaneous melanoma [51].

## 4. MicroRNAs as Tissue-Based Melanoma Markers

By analyzing formalin-fixed paraffin-embedded (FFPE) samples, Babapoor et al. [52] discovered that the expression of miR-211 was increased in the common and dysplastic nevi groups, but decreased in the melanoma samples. With 90% sensitivity and 86.2% specificity, in situ hybridization of miR-211 assisted in differentiating melanoma from the nevus group [52]. Sahranavardfard et al. [53] measured the expression of miR-203 in melanoma stem cells and indicated a potential function in enhancing melanoma cell proliferation and growth, which was linked to the upregulation of BRAF. Nevertheless, miR-203 expression was downregulated in tissue samples. While there is a need for more research on the role of miR-203 in tumor growth, Sahranavardfard et al. proposed that miR-203 might be used as a diagnostic biomarker for melanoma metastasis [53]. MiR-378 was shown to be highly expressed in melanoma tissue samples, according to Sun et al. [54]. Its inhibitory action on the FOX3 target gene via the Wnt/-catenin pathway stimulated the proliferation and migration of melanoma cells in tissue samples, suggesting that it could be used as a diagnostic biomarker for metastasis [54].

A summary of the described microRNA molecules, including both the blood-based as well as tissue-based melanoma markers, is presented in the table below (Table 1).

## 5. MicroRNA as a Potential Biomarker to Detect Cutaneous Malignant Melanoma

**Figure 1:**
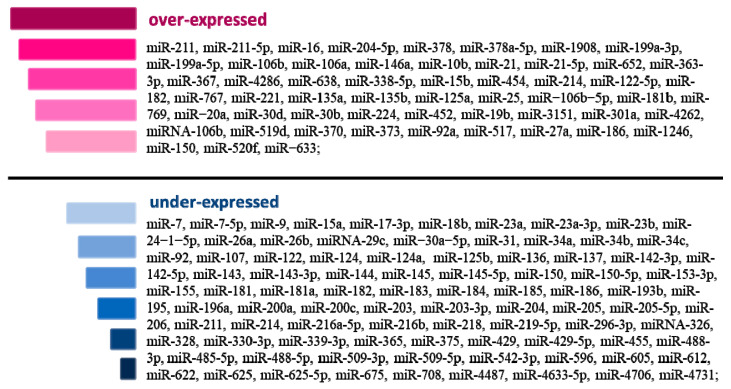
MicroRNAs in CMM melanoma. The figure shows lists of under-expressed and over-expressed miRNAs in cutaneous malignant melanoma.

MicroRNAs have been shown to regulate oncogenesis and different steps of cancer progression including melanoma [10,56]. Since miRNA was found to be detectable in extra- and intracellular environments, all eyes of medical science have turned to miRNAs as new potential biomarkers. Many studies have indicated that circulating miRNAs may come directly from the cancer tissue and may indicate tumor progression. Interestingly, after tumor resection, levels of these miRNAs tend to decrease [57]. There have been doubts for some time about the persistence of miRNA as a potential biomarker because of the elevated levels of nucleases found in plasma [58], but the results showed stable miRNAs in the fixed tissues samples and dispelled all the doubts. There were several reported between different teams, which had analyzed the same tumors and had divergent results [59]. The reason for this could be that there were different conditions during sample collection, storage, transport, analysis, etc. Therefore, to solve this problem, protocols must be standardized and developed for the initial stages of the sample collection process and the methods for analyzing the results. Currently, miRNA as a biomarker can be determined in cancer patient tissue, such as primary tumor or metastases tumor after surgery, or in body fluids (mainly arterial or venous plasma and serum), with no significant difference. Depending on the type of cancer, the expression of various abnormal miRNAs correlates with tumor classification, stage, prognosis and likely response to therapy. Below, we present selected miRNAs that meet the requirements as potential biomarkers in the clinical setting, i.e., appropriate in specific cancer type, significantly differentially expressed and, of course, in correlation with the patient’s condition [56]. The list of reported over-expressed and under-expressed miRNAs in melanoma are presented in Figure 1.

## 6. Examples of Melanoma-Related MicroRNAs

**Figure 2:**
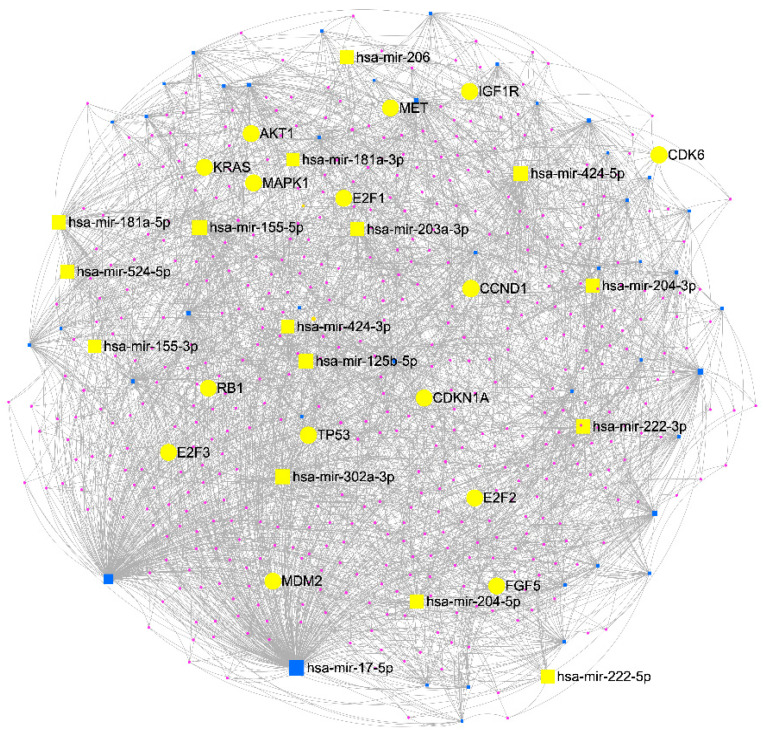
miRNA network analysis interaction with genes in CMM melanoma. The miRNA-target gene interaction network containing 550 nodes and 2619 edges. Squares indicate miRNA examples, yellow squares indicate miRNAs related to melanoma and yellow circles are the interacting genes in melanoma. The visualization was performed using miRNet 2.0 [60].

New examples of microRNAs associated with the development and progression of melanoma are known. Of course, some of the microRNA molecules are highly associated with the expression of genes directly related to the development of melanoma. Using the available databases, we have prepared Figure 2 below, which shows the interaction of microRNA with genes related to melanoma. The visualization was performed using miRNet 2.0 [60]. Below we present and describe examples of microRNAs that are related to CMM melanoma. Using bioinformatic analyses, such as Cytoscape, miRNET and others, we could identify potentially crucial miRNA-related genes and key pathways in cutaneous malignant melanoma. It has been shown that the use of an algorithm based on a network of molecular targets with genes and pathways has great potential in the detection of potential biomarkers. From Figure 2, we see that there are several miRNA molecules and genes that deserve a closer look in future research.

The involvement of miR-181a/b as a significant factor in the drug resistance of melanoma was analyzed by Barbato et al. [61] by identifying changes of expression in melanoma cell lines sensitive and resistant to the BRAF inhibitor dabrafenib. Upregulated expression of miR-181a/b was present in BRAFi-sensitive cells. The repression of miR-181a/b induced BRAFi sensitivity in previously resistant cells. The data indicated that by manipulating the activation of mir-181a/b, resistance to BRAFi could be regulated. Additionally, in the cohort of 16 patients, the upregulated expression of miR-181a/b was positively correlated with better survival of patients. Finally, the study showed in 17 melanoma samples with the V600 BRAF mutation that resistance to BRAFi and suppression of tumor growth was induced by miR-181a/b targeting TFAM and inhibiting its expression. In conclusion, miRNA-181a and miRNA-181b could serve as potential biomarker to measure patients survivability in melanoma and define melanoma cells responsiveness to the administrated drugs.

The proliferation and migration of melanoma cells could be affected by miR-633. Upregulated levels of miR-633 were negatively correlated with KAI1 expression in melanoma tissue samples. By transfecting cells with inhibitor of miR-633, cell proliferation and migration were suppressed. MiR-633 may serve as a potential candidate for the diagnosis and treatment of human melanoma [62].

The role of miR-128-3p as a tumor growth suppressor was studied by Zhou et al. [63]. In melanoma cells miR-128-3p was downregulated. The overexpression of miR-128-3p suppressed the apoptosis, proliferation, invasion and migration of melanoma cells. NTRK3, which was measured to have an upregulated level in melanoma cells, was directly targeted and its expression inhibited by miR-128-3p. Additionally, downregulating NTRK3 resulted in enhanced apoptosis and a reduction of the proliferative and invasive features. In conclusion, NTRK3, which serves as an enhancer of the malignant phenotype, could be a new target and novel biomarker for future therapies against melanoma. These treatments could focus on significantly upregulating the expression of miR-128-3p, which is proven to suppress NTRK3 carcinogenesis.

The effects of miR-137 were studied by Zhang et al. [64] in uveal melanoma. In UM tissues there was reported to be an upregulated level of miR-137 in comparison to control non-tumorous uveal tissues. MiR-137 upregulation through mimics transfection in melanoma cells dramatically impaired the proliferation. On the contrary, miR-137 inhibition promoted melanoma cell viability. In tissue transfected with miR-137 melanoma cells, there were restrained invasive and migratory features of melanoma cells. Luciferase activity of EZH2-3′UTR-MT confirmed that miR-137 targeted EZH2, which regulated Wnt/β-catenin and EMT. Data indicated that the miR-137/EZH2 axis could be a new target for UM therapy.

The function of miR-18a-5p in melanoma progression and pathogenesis was studied in melanoma and skin tissues from 20 patients with malignant melanoma [65]. High levels of miR-18a-5p in melanoma tissues indicated its pro-oncogenic influence in melanoma. Moreover, inhibiting miR-18a-5p slightly reduced the proliferation of melanoma cells compared with a control group. Furthermore, a negative correlation was discovered between miR-18a-5p and EPHA7, through the inhibition of EPHA7 by binding miR-18a-5p. Regarding the malignant phenotype inhibition of EPHA7 by miR-18a-5p, it promoted the proliferation, autophagy and blocked apoptosis of melanoma cells. These results indicate that the role of miRNA can be further studied in order to find new melanoma biomarkers and therapeutic methods.

The function of miR-524-5p was investigated as a modulating factor of tumor growth and BRAFi resistance. In one study [66], 207 tissues were used. In normal skin tissue, mir-524-5p was more expressed than in melanoma tissue. Moreover, nevus tissue had a higher expression of miR-524-5 than melanoma tissue. It was observed that miR-524-5p may be used as a prognostic biomarker in PLX4032 (BRAFV600E inhibitor) treatment, in which patients with a higher expression of miR-524-5p were associated with a better response to this therapy. By analyzing melanoma cells resistant to BRAFi, proliferation was suppressed and, additionally, the overexpression of miR-524-5p inhibited the migration and invasion of resistant cells. MiR-524-5p was an inhibitor of the MAPK/ERK pathway and partially blocked the PI3K/AKT signaling. Data showed that modulating the miR-524-5p expression could stop BRAFi-resistant melanoma progression and indicated the miR-524-5p is a potential biomarker of response to BRAFi therapy.

The influence of miR-4458 on melanoma tumorigenesis was studied by Zhou et al. [67]. By transfecting miR-4458 mimics, it was shown that melanoma cells’ malignant features were inhibited and apoptosis was promoted by overexpression of miR-4458. MiR-4458 tumor inhibition features occurred via binding with PBX3 3′-UTR. The tumor-suppressing effect of miR-458 was suppressed through the upregulation of PBX3. Mediating MiR-4458/PBX3 could constitute a new target for melanoma therapy.

In 2016, Li et al. [68] measured the expression of miR-137 in patients with cutaneous melanoma (CM) and found a correlation between miR-137 and the prognosis of CM patients. The relative expression of miR-137 in CM tissue was significantly lower compared to normal tissue. A Chi-square analysis showed statistical significance between miR-137 expression and clinical characteristics—TNM classification of malignant tumors stage. The survival curve indicated that patients with low expression of miR-137 showed higher mortality, suggesting that low miR-137 expression indicated a poor prognosis for CM melanoma patients.

The aim of Xu et al.’s [69] study was to determine the clinical value of miR-424 in melanoma. They demonstrated that the expression of miR-424 remarkably increased in tissues and serum of patients with melanoma. Moreover, the ROC (receiver operating characteristic) analysis showed that the expression of miR-424 in tissue and serum can serve as a diagnostic biomarker for melanoma. The expression of miR-424 was correlated with tumor thickness, metastasis and tumor stage. In their project, patients with higher miR-424 expression showed decreased overall survival (OS) and disease-free survival (DFS), so they postulated that high miR-424 expression would result in a poor prognosis for melanoma patients.

In 2015, the aim of the Wang et al. [70] study was to determine the expression and prognostic role of miR-203 in melanoma patients. A significant alteration of miR-203 in a number of cancers was previously reported. Wang et al. showed decreased expression of miR-203 in melanoma tissues compared with non-cancerous tissues, which is important from a clinical point of view, as the downregulation of miR-203 was associated with tumor thickness and tumor stage, and a Kaplan–Meier analysis revealed that low miR-203 expression was correlated with short overall survival (OS) of patients. The analysis also indicated that miR-203 could be an independent prognostic marker in melanoma, or could even become a new therapeutic target for melanoma patients.

MicroRNA-106b (miR-106b), another potential biomarker of melanoma, is overexpressed in many types of cancers and is associated with processes of carcinogenesis. Its clinical significance in cutaneous melanoma was reported by the study of Lin et al. [71]. They postulated that high miR-106b expression is associated with Breslow thickness, tumor ulceration and an advanced clinical stage. Patients with high expression of miR-106b had a shorter 5-year overall survival (OS). Their analysis showed that miR-106b can be an independent prognostic factor for the OS of melanoma patients (associated with poor prognosis) and may be related to cutaneous melanoma progression. These results suggest that miR-106b is a promising biological marker for cutaneous melanoma.

The next miRNA tumor suppressor that has been implicated in the aggressive progression of cancer is miR-206. In 2015, Tian et al. [72] presented evidence that miR-206 was correlated with the aggressive progression of melanoma. The expression levels of miR-206 in melanoma serum samples were significantly lower than in healthy controls and a low level of miR-206 was observed especially in patients with two or more metastatic sites (higher clinical stage). Patients with low serum miR-206 levels also had a significantly shorter 5-year overall and disease-free survival than those with high serum miR-206 levels.

MiR-222 was studied by Lionetti et al. [73] in human IGFR39 melanoma cells. In the study, the mathematical model of phenotypic switching cancer cells to cancer stem cells (CSC) was applied and experiments were conducted, which explained that switching into cancer stem cells is controlled by MicroRNA-222 in CSC. IGFR39, after the reduction of hsa-mir222-5p activity, could not weaken the activity of CSC’s markers and switched to a less malignant phenotype. Knowing the factor of phenotypic switching into CSC, the inhibition of hsa-mir222-5p can be used as a therapeutic option method in melanoma cancer patients. MiRNA-222 is a factor affecting the increase in melanoma malignancy. By upregulating the level of the PI3K/AKT pathway, it was shown that miRNA-22 within exosomes could be absorbed by recipient melanomas. The enrichment of miRNA-22 in cancer cells was correlated with the downregulation of targeted gene p27Kip1 and increased vascularization of the tumor. The result of the study was then confirmed by administrating miR-222 antagomir-carrying exosomes that caused upregulation of the previously inhibited gene and slowed the mitosis cycle rate and reduction of cell growth factors. The study suggested that miR-222, together with its exosome-associated features, can be used as a diagnostic biomarker for melanoma and constitutes a potential target for future immunotherapy.

MiR-125a-5a was investigated as a drug resistance factor to BRAF. By profiling genes for BRAF resistance, an upregulation of MiR-125a-5a was found in BRAF-resistant cells. Secondly, the study showed that cells with overexpression of MiR-125a-5a could avoid apoptosis through the targeting of proapoptotic factors MLK3 and BAK by miR-125a. This finding indicates that the therapeutic method of blocking microRNA-125a can revert the resistance of BRAFV600E melanoma and be successfully treated with BRAF inhibitor [74].

Mir222-5p was analyzed as an important factor in melanoma progression, resistance against BRAF and as a prognostic factor. Rambow et al. [75] analyzed the influence of the TCF4/miR-125b/NEDD9 cascade on melanoma progression. Their results showed that overexpression of miR-125b was present in metastases. Moreover, high activity of miR-125b indicated the pro-invasive an pro-migrating character of melanoma. Knowing the mechanism through which melanoma gains its invasive feature, this can be used to stop certain elements of this cascade and ensure a longer life for patients with melanoma. It was proven by Nyholm et al. [76] that miR-125b reduces the proliferation focally and induces senescence in human melanoma cells, which could be useful in future therapies. Moreover, it was confirmed that miR-125b acts as a tumor suppressor by knocking down the expression of c-Jun factor, which is responsible for the cell cycle [30]. Another factor targeted by miR-125b-5p is MLK3. Zhang et al. [77] measured high MLK3 levels in metastatic melanoma and showed that activity of MLK3 is reduced by artificially overexpressing miR-125b-5p. Vergani et al. reported that melanoma resistance to vemurafenib was linked to CCL2 [78]. In their study, the authors measured high CCL2 expression levels in melanoma cells and plasma of patients after vemurafenib treatment. Apoptosis from BRAF inhibitors was correlated with high production of autocrine CCL2. High levels of CCL2 were parallel with high expression of miR-34a, miR-100 and miR-125b. Yan et al. [79] stated the importance of microRNA-125b as a prognostic factor in melanoma by measuring the expression of microRNA-125b in formalin-fixed paraffin-embedded (FFPE) melanoma tissues. The expression of microRNA-125b was reported to be low in lymph node metastasis of melanoma and metastasis, as reported in previously mentioned studies. The group of subjects with high miR-125b expression levels was characterized with lower risk of death than the group with lower miR-125b expression. Thus, miR-125b could be a significant prognostic factor and biomarker of melanoma malignancy, and a potential target for melanoma treatment.

99a/let-7c/125b-2 was studied in terms of its targeted expression in melanoma phenotypic changes. In invasive cell lines, miR-99a and miR-125b were highly expressed, in contrast to let-7c, which was overexpressed in proliferative cell lines. Additionally, melanoma cells with a let7-c mimic were less invasive than the control group. In conclusion, these results confirmed that the invasiveness and proliferation of melanoma can be influenced by controlling expression of the 99a/let-7c/125b-2 cluster. Moreover, miRNA mimics or antagonists could be used as a therapy method to reduce the risk of metastasis of melanoma. In diagnostics, by measuring the expression of miRNA we could determine if melanoma is in its invasive stage [80].

MiR-106b-5p was investigated as a potential factor controlling the cell cycle via targeted PTEN expression. The quantity of MiR-106b-5b was high in malignant melanoma tissue and by downregulating PTEN it activated the Akt/ERK1/2 signaling pathway, which promoted MM cells proliferation. Thus, MiR-106b-5p might be a target in future cellular therapies in order to stop MM progression [81]. MiR-106b-5p could be used as a prognostic factor and indicator of future melanoma progression. In malignant melanoma cells, the expression of MiR-106b-5p was significantly higher than in control human epidermal melanocytes. MM cells with highly expressed MiR-106b-5p could secrete MiR-106b-5p exosomes to melanocytes. Increasing the level of MiR-106b-5p promotes invasion and migration of transfected cells by blocking EphA4. Blocking EphA4 resulted in activation of the ERK1/2 pathway, which promoted metastasis in vivo [82]. Moreover, high expression of MiR-106b-5p was correlated with lower patient survival [71]. Regarding cell proliferation, blocking miR-106b-5p causes inhibition of the cell cycle by blocking p21/WAF1/Cip1 and lowers levels of cyclins and CDKs proteins. Furthermore, by treating melanoma cells with grape seed proanthocyanidins (GSPs), the expression of miR-106b-5p was downregulated and p21/WAF1/Cip1 was reactivated, which stopped proliferation [83]. Results have shown that a high expression of MiR-106b-5p induces epithelial–mesenchymal transition of MM cells, which causes progression and metastasis. A new therapy method using grape seed proanthocyanidins can be conducted, which stops melanoma proliferation by lowering levels of miR-106b-5p.

MiR-155 has been widely studied as a regulating factor in antitumor responses of T lymphocytes and a regulator of myeloid-derived suppressor cells (MDSCs) activity in malignant melanoma. The transition of CD14+ monocytes into MDSCs (CD14 + HLA-DRneg cells) was induced in vitro by secreted extracellular vesicles from melanoma cells. In extracellular vesicles, miR-146a, miR-155, miR-125b, miR-100, let-7e, miR-125a, miR-146b and miR-99b were highly expressed. Blocking miR activity led to a reversion of EV–MDSC changes. In plasma from 20 patients with an advanced melanoma stage there were significantly higher levels of all MDSC-miRs than in healthy donors, and high MDSC-miRs expression was associated with lower overall survival [84]. In a study by Wang et al. [85], melanoma progression was significantly faster when suppressing the expression of miR-155. Low levels of miR-155 caused increased migration of myeloid-derived suppressor cells (MDSCs) in mouse tumor tissue, spleen and marrow via increased expression of HIF-1α mRNA. Accumulated MDSCs contributed to a lower number of T cells and provoked angiogenesis in comparison to the control group. This could be a mechanism of tumor progression due to the immunosuppressive abilities of MDSCs [86]. It was reported by Martinez-Usatorre [87] that T-cell stimulation levels were positively correlated with the expression level of miR-155. Moreover, miR-155 expression was associated with the avidity of CD8+ T-cell towards melanoma cells and low expression of its targeted genes was associated with prolonged survivability. The influence of miR-155 on T cells’ antitumor response was also confirmed by Huffaker et al. [88]. High levels of miR-155 in melanoma were associated with a proper antitumor T-cell response. By downregulating miR-155 in melanoma cells, the quantity of CD4+, CD8+ T cells and IFNγ+ levels decreased, which resulted in an increase in tumor size. By providing αPD-1, αPD-L1 and αCTLA-4 antibodies in cells without miR-155 expression, numbers of CD4+, CD8+ T cells and IFNγ+ increased. In conclusion, αPD-1, αPD-L1 and αCTLA-4 antibodies could be used to provoke an antitumor T-cell response against melanoma. Overexpression of miR-155 in T cells could enhance could ensure a better treatment outcome against melanoma cells. Furthermore, along with expression levels of miR-155 the survivability rate of patients can be assessed. Apart from an immunological affinity in melanoma, miR-155 can affect other cell responses. Exosomes secreted by melanoma cells to fibroblasts are contained within miR-155, which activates the JAK2/STAT3 signaling pathway and suppresses expression of the SOCS1 gene. The whole process causes a proangiogenic effect in melanoma [89]. A study confirmed that high expression of miR-155 in 49 borderline melanocytic lesions and dysplastic naevi was associated with the depth of lesions. Melanocytic lesions with a higher mitoses rate were characterized by an upregulated expression of miR-21 [90]. MiR-155, by targeting SKI mRNA and downregulating SKI mRNA translation, also blocks the proliferation of melanoma cells. However, the SKI mechanism was not a leading factor in regulating cell proliferation, because in an experiment miR-155 was characterized with a greater growth-suppressing effect than SKI alone [91]. Besides proliferation, miR-155 is involved in inflammatory pathways in melanoma cell lines. In the study by Arts et al., Interleukin-1ß promoted expression of miR-155. Then based on a mouse model, it was proven that upregulated levels of miR-155 induced repression of MITF-M, which indicated an anti-inflammatory effect of miR-155 induced by Il-1ß. New methods in melanoma treatment could be developed by blocking miR-155 and reducing inflammation [92].

MiR-17-5p was studied as a potential factor modulating PD-L1 mRNA expression in BRAFi-resistant melanoma cells. A correlation was reported between repressed miR-17-5p and the upregulated level of PD-L1 mRNA, and it was proven that miR-17-5p directly binds to the 3′-UTR region of PD-L1. In a group of BRAF-treated patients with melanoma, those with PD-L1+ were characterized with the worst prognosis. Moreover, the quantity of miR-17-5p was higher in PD-L1+ than PD-L1- tissue lesions and BRAFi resistance increased the expression level of PD-L1. In conclusion, expression of PD-L1 is negatively correlated with miR-17-5p. Since high expression of PD-L1 was associated in the study with invasiveness and aggressiveness, measuring miR-17-5p can be used as a predictive factor of melanoma treatment and progress [93].

The role of miR-138-5p as a growth suppressor in melanoma was investigated in the study by Tarazón et al. [94]. Their results showed that MiR-138-5p targeted human telomerase reverse transcriptase gene (hTERT) and reduced its translation. The upregulation of MiR-138-5p stopped cell proliferation on the G0/G1 phase. Providing miR-138-5b in future therapies could be effective in melanoma treatment.

MiR-1180-3p could be used as a diagnostic marker in melanoma. Lowered expression of miR-1180-3p was present in exosomes extracted from melanoma with greater invasive and proliferation features than melanoma with high levels of miR-1180-3p. By proceeding with knockdown and upregulation of miR-1180-3p, a negative correlation was confirmed between mir-1180-3p and its targeted genes MAN2B1 and ST3GAL4. An upregulated level of miR-1180-3p marginally increased expression of MAN2C1, which was not significantly affected by the miR-1180-3p inhibitor. A lowered migration ratio was observed in ST3GaL4-deficient melanoma cells. Moderating the activity of miR-1180-3p and ST3GAL4 can be used as a therapeutic method in melanoma. Moreover, as a future diagnostic marker, low expression of miR-1180-3p derived from melanoma cells can indicate a more aggressive melanoma state [49].

MiR-10a-5p was studied as an inhibiting factor in melanoma progression. A reduction of miR-10a-5p was associated with better overall survivability. By targeting LIN28B I in melanoma cells its malignant phenotype could be blocked in humans and mice. These changes could be reverted by inducing LIN28B expression. MiR-10a-5p is directly regulated by TCF21, which binds to its promoter region. The TCF21/miR-10a-5p/LIN28B pathway could be targeted by a repressor in a new melanoma treatment in order to stop melanoma progression [95].

MiR-302a-3p was investigated as an inhibitor factor of METTL3 expression in melanoma. In the group of 35 human primary melanomas and 35 metastasis melanomas it was proven that METTL3 promotes the invasion and migration of melanoma cells. miR-302a-3p was responsible for the upstream regulation of METTL3 in melanoma. A negative correlation between METTL3 and miR-302a-3p was observed. MiR-302a-3p, by blocking the expression of METTL3, reduced tumor growth metastasis and other malignant behavior features. The study found a new target, the miR-302a-3p/METTL3 axis, for future melanoma therapies [96].

The suppressive role of MiR-485-5p in melanoma proliferation was explored in a study by Wu et al. [97] in a group of 55 human melanomas from patients. Their results showed that in melanoma cells PRRX1 was overexpressed, and it was regulated by miR-485-5p. Additionally, a negative correlation between upregulated PRRX1 and downregulated miR-485-5p was confirmed. In melanoma cells with transfected mirR-485-5p, by measuring luciferase activity it was shown that miR-485-5p repressed PRRX1, which blocked the TGFβ pathway and EMT. Overexpression of miR-485-5p reduced the ability of melanoma cells to proliferate and weakened its invasive features. These results suggest a possibility of utilizing the tumor suppressing features of miR-485-5p as a novel therapy or biomarker for melanoma progression.

MiR-34a and its role as a tumor suppressor was analyzed in the context of inhibition of ZEB1. By comparing the expression data from melanoma and control tissue, downregulation of miR-34a was present and correlated with TNM stage of melanoma. In comparison to miR-34a, expression of ZEB1 was significantly higher than in the control. Overexpression of miR-34a suppressed the cell proliferation and migration via targeting ZEB1. In vivo miR-34a significantly inhibited tumor growth and reduced ZEB1 expression. These results indicated that mir34a is a potential target for new melanoma treatment [98].

The influence of BRAFI resistance on miR-129-5p expression in melanoma was studied by Gebhardt et al. [99]. By providing vemurafenib to sensitive melanoma cell lines, expression of miR-129-5 was induced. In comparison to sensitive cell lines, BRAF-resistant cell lines were not characterized by a shift in miR-129-5p expression. MiR-129-5p expression was increased in a span of 20 days of vemurafenib treatment, but miR-129-5p expression was inhibited in BRAF-resistant melanoma cells with vemurafenib. It was proven that MiR-129-5 expression was influenced by the BRAF/MEK pathway and inhibited by EZH2. Additionally, MiR-129-5p acted as a tumor growth suppressor in melanoma cells. Moreover, it was reported that miR-129-5p binded with the SOX4 gene, which induced BRAFi sensitivity in melanoma cells. In conclusion, the upregulation of miR-129-5p, as well as the blocking of SOX4, could be used to ensure better treatment outcomes in melanoma BRAFi-resistant cells.

In treatment of melanoma with hydatid cyst fluid (HCF)-based therapeutics, the expression of miR-365 was affected, which was studied as a tumor apoptosis factor. HCF significantly increased the expression of miR-365, which inhibited melanoma growth by activating pro-apoptotic Bax, Caspase-9 and Caspase-3, which are apoptosis-inducing factors. Echinococcus fluids and its stimulating effect on miR-365 expression could be a valuable element of future drugs that would work against melanoma progression [100].

MiR-942-5p was studied in melanoma cell lines to find its effect on cell proliferation and melanoma progression [101]. In melanoma cells, the level of miR-942-5p was upregulated. Its overexpression reduced the number of apoptotic cells and promoted cell proliferation, invasion and migration. The results indicated that miR-942-5p targets 3′-UTR of DKK3. Additionally, DKK3 protein levels were lowered by transfecting miR-942-5p mimics and increased by miR-942-5p inhibitor. Moreover, b-catenin expression in cytoplasm and the nucleus increased after transfection with miR-942-5p mimics. In conclusion, the results indicated the possibility of miR-942-5p use as a potential diagnosis and biomarker in melanoma. Moreover, future drugs against melanoma progression could focus on targeting the DKK3 and Wnt/b-catenin pathway.

MiR-147a is involved in modulating the malignant phenotype through the hnRNPK/LINC00263 pathway in malignant melanoma. The upregulation of MiR-147a weakened its invasive features and blocking of miR-147a caused the opposite effect. miR-147a targeted the CAPN2-controlled proliferation rate and invasion. It was additionally found [102] that miR-147a directly binds to LINC00263, which functions as a competitive endogenous RNA (ceRNA) for other tumor suppressive miRNA. Thus, the suppressive action of miR-147a on melanoma progression could be negatively altered through the stimulation of LINC00263 expression, which would lead to the upregulation of CAPN2-promoting melanoma proliferation.

MiR-548b and its targeted HMGB1 gene suppressed melanoma growth, migration and invasion in melanoma tissues. In melanoma, miR-548b expression was observed to be downregulated compared to adjacent tissue and overexpression of miR-548b indicated better overall survivability in melanoma patients [103]. MiR-548b, by targeting the HMGB1 gene, suppressed melanoma cell migration and invasiveness in vitro. In conclusion, miR-548b, through inhibiting the expression of HMGB1, significantly contributes to melanoma suppression.

In the study by Zhao et al. [104], miR-675-3p was identified to be overexpressed in melanoma cell lines, tissues and blood, and may play a major role in melanoma progression. Therefore, the correlation was assessed between miR-675-3p and tumor phenotype. Tumor histologic grade and Clark’s level was accompanied with high miR-675-3p levels. Other features, such as TNM stage, age and gender, were not correlated with expression. Their study confirmed that miR-675-3p binded with OPCML, which significantly downregulated its expression. Regarding cell cycles, a positive correlation was reported between the TGF-β/SMAD, HIF-1 signaling pathways and miR-675-3p. These results indicated that miR-675-3p functions as a mediator of many cellular pathways in melanoma, which indicates its usefulness as a cancer biomarker.

MiR-7013-3p was investigated by Huang et al. [105], with a focus on its overexpression during Hydroxyurea (HU) treatment in melanoma cells was. After HU treatment miR-7013-3p targeted the MITF 3’UTR gene and, thus, negatively controlled its expression. Transfecting melanoma cells with miR-7013-3p stimulated apoptosis, but caused a reduction in the proliferation and migration of melanoma cells. The data showed that an upregulated miR-7013-3p level serves as an antitumor factor in melanoma and it indicates the positive effects of HU on melanoma progression.

The immune modulatory role of MiR-200a-5p was investigated. Upregulation of miR-200a-5p, which targeted the TAP1 3′-UTR, caused the downregulation of TAP1 protein [106]. The suppression of TAP1 protein resulted in downregulation of HLA1 surface expression on melanoma cell lines. Moreover, there was a positive correlation between high expression of TAP1, HLA-A mRNA and survivability. Patients who exhibited low levels of TAP1 a had low quantity of immune cell subpopulations in comparison to the high level TAP1 group with more immune cell populations. Worse overall survivability in metastatic melanoma was connected with upregulation of miR-200a. In conclusion, miR-200a-5p could be used as a new target in melanoma treatment or to distinguish which patients might respond better to immunotherapy.

MiR-122 and miR-144 were analyzed by Amaro et al. [107] for their tumor suppressive roles in uveal melanoma. In uveal melanoma cell lines low expression of miR-122 and miR-144 was observed and this was associated with higher expression of ADAM10 and c-Met. As a result, miR transfection inhibited the expression of ADAM10 and c-Met protein by binding directly to the 3′UTR region of both ADAM10 and c-Met. Moreover, transfection reduced cell proliferation and migration, but increased apoptosis. Knowing the molecular mechanism of miR-122 and miR-144’s suppressive control of melanoma cells could be used in future uveal melanoma treatment.

MiRNA-145/miRNA-205 is another suppressive factor influencing gene expression in highly invasive UM and less invasive UM. In comparison to a healthy control, more invasive and less invasive UM were characterized by lower expression of miR-145. Additionally, the level of miR-205 was measured to be lower in the healthy control and significantly lower in the UM group with the more invasive phenotype. Overexpression of miRNA-145 and miRNA-205 through transfection of miR mimics showed a suppression of proliferation and invasiveness. The mechanism of miRNA-145/miRNA-205 suppression was based on the inhibition of NRP1 expression and, thus, downregulation of EMT-related protein CDC42 (NRP1 is co-expressed with CDC42 in the UM). In conclusion, the effect of miRNA-145 and miRNA-205 could restrain melanoma’s invasive features. MiRNA-145 and miR-205 downregulation of EMT-related protein CDC42 through the blocking of its NRP1 gene could be used in future treatment of UM [108].

MiR-152-5p is a potential regulator of drug resistance and metastasis in melanoma [109]. In BRAFi-resistant cell lines, the expression of miR-152-5 was significantly increased. Mir-152-5p promoted metastasis and invasive features of BRAFi-resistant melanoma by blocking metastasis suppressor gene TXNIP. Overexpression of TXNIP turned out to be a major inhibiting factor for cell migration and invasion in BRAFi-resistant melanoma cells. Moreover, in BRAFi-resistant cells after vemurafenib treatment, demethylation of miR-152-5p promoter occurred, which resulted in its activation. The results suggests that blocking miR-152-5p could possibly slow down the process of acquiring BRAFi-induced resistance. Discontinuous BRAFi administration could also stop the switch to a more resistant and invasive phenotype of BRAFi-resistant melanoma.

The miRNA-183 cluster plays an important role in tumor proliferation in melanoma cell lines [59,110]. By measuring luciferase activity, it was shown that the upregulated miR-183 cluster targeted MITF, reducing its expression and causing a reduction in melanin production. Moreover, the MEK/ERK pathway was modulated by miRNA-183, which was observed in cells with overexpressed miR-183 and reduced cell proliferation rate. The results showed that miRNA-183 could be a new prognostic predictor and a novel target for melanoma treatment.

The influence of overexpressed miR-652 on tumor development in uveal melanoma was studied by Xia et al. [111]. Inhibition of miR-652 caused tumor suppression by reducing cell migration and proliferation. MiR-652 suppressed the expression of HOXA9, which activates the HIF-1 signaling pathway, affecting melanoma suppression. Additionally, a negative correlation was observed between miR-652 and HOXA9 mRNA levels in tumors. By targeting the miR-652/HOXA9/HIF-1 a suppressive effect on UV progression could be obtained, which would be beneficial in future UV treatment.

MiR-25, miR-204, miR-211, miR-510 and miR-513c were confirmed to be prognostic factors, through data evaluated from 80 upregulated and 105 downregulated miRNAs from primary and metastatic melanomas [112]. There was a connection found between T stage, Breslow depth value and all of the miRNAs except miR-25. The prognosis was positively correlated with miR-204, while other miRNAs were negatively correlated. Moreover, there was possible involvement of these miRNAs in PI3K-Akt pathways, ubiquitin-mediated proteolysis and focal adhesion, but further research needs to be conducted to find the nature of the involvement mechanisms of MiR-25, miR-204, miR-211, miR-510 and miR-513c.

The correlation between miR-214 and the Wnt/β-catenin pathway was studied in melanoma cells [113]. MiR-214 targets the 3′UTR of β-catenin, and miR-214 overexpression caused by transfecting cells with miR-214 mimics resulted in the reduction of β-catenin mRNA levels; however, no decrease in β-catenin protein levels was observed and, furthermore, there was no correlation between the quantity of miR-214 and β-catenin protein expression. Regarding melanoma progression and treatment, overexpression of miR-214 promoted cell proliferation and affected BRAFi sensitivity by reducing the number of cells killed. Analyzed data of 199 candidate genes showed that the majority of targeted genes belonged to the Wnt pathway. In conclusion, MiR-214 regulates the Wnt signaling pathway primarily by modulating its negative regulators ankyrin repeat domain 6 (ANKRD6) and C-terminal-binding protein 1 (CTBP1). MAPK treatments against melanoma could be more effective by overexpressing miR-214 target proteins in order to enhance sensitivity to MAPKi.

MiR-300 could play a key role in the pathogenesis of melanoma formation. In a mouse model, out of 73 genes involved in melanocyte progression to melanoma cells after a UV dose, there was inhibition of GADD45B [114]. MiR-300, which binds to the 3′-UTR of GADD45B, was increased only as a result of UV exposure, a response to DNA damage. The upregulation of miR-300, MYC, PPARG and ZIC2 was observed in melanoma cells. MiR-330/GADD45B was reported interact with TP53, JUN, JUNB, FOS and FOSB, which could possibly affect melanoma progression and might worsen the prognosis. These results presented a TFs–mRNA–miRNA axis in melanoma cells, which in further research could be established as a new potential therapeutic target against melanoma.

In the context of melanoma metastasis, an evaluation of expression data of various miRNAs shed light on the function of particular miRNAs in melanoma metastasis. It was stated that miR-155-5p, miR-18a-5p and miR-93-5p participate in the development of metastasis. MiR-93-5p was verified as a pro-invasive miRNA through mediating UBC expression, which could be considered a potential target of inhibition in future treatments of melanoma [115]. The main challenge for scientists and physicians is to discover specific miRNAs for melanoma as biomarkers in a broad range of patients, enabling the development of a simple, accurate and inexpensive method of diagnosis or treatment. Not only miRNAs, but also other non-coding RNAs (ncRNAs), such as long non-coding RNAs (lncRNAs), small nucleolar RNAs (snoRNAs) and circular endogenous RNAs (ceRNAs), could have potential use as biomarkers in cancer diagnosis and prognosis [116].

## 7. MicroRNA as a Drug in Clinical Treatment

**Figure 3:**
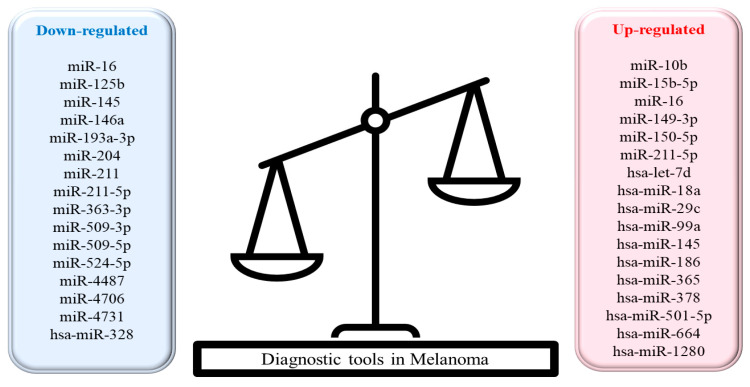
MicroRNAs as diagnostic tools in CMM melanoma. The figure shows the application of downregulated and upregulated miRNAs that can be used as diagnostic tools in cutaneous malignant melanoma.

It is likely that miRNAs are one of the most commonly used groups of RNAs in clinical treatment. A typical miRNA is about 22 nucleotides long and is converted from a long primary RNA sequence to a short and functional transcript. A characteristic of miRNAs is their ability to target hundreds or even thousands of genes [117]. Today, great hopes are placed on therapeutic drugs with small RNA (less than 200 nucleotides long). One reason is that in 2018, the Food and Drug Administration (FDA) approved the first small interfering RNA (siRNA) for the treatment of polyneuropathy caused by hereditary transthyretin-dependent amyloidosis (hATTR) [118,119]. MiRNAs are deregulated in almost all malignancies. Specific miRNA expression profiles allow the characterization of different cancers and stages, so miRNAs can also be used in therapy and diagnosis of cancer patients [119]. However, despite initial successes, this use of miRNAs is a new therapeutic modality that is proving to be challenging in the context of design and efficacy [120]. RNA oligonucleotides have characteristics that can complicate these processes, which include degradation by nucleases when added to biological systems, poor cell-membrane penetration, endosome entrapment, poor binding affinity to complementary sequences, poor delivery to desired target tissues, off-target and unwanted toxicity and activation of innate immune responses [121,122,123,124,125,126]. However, despite the difficulties, research is being conducted to solve these problems. The chemical modification of miRNAs on the phosphodiester backbone and 2′ of the ribose is a proposal to improve the stability of miRNAs to protect them from degradation. Naked miRNAs with unmodified 2′OH in the ribose moiety are degraded within seconds by nucleases, such as RNase A-type nucleases in blood serum, and are removed very rapidly by the kidney, which contributes to their short half-life in the systemic circulation. Modifications, such as phosphodiester bonds or ribose backbone, also improve the binding affinity to the target [127,128]. Another problem of efficient delivery of miRNAs to the target tissue is addressed by introducing different carriers [120]. For this purpose, nanocarriers (liposomes) or polymers using leaky tumor vessels are used (conjugation-based methods in which a sugar, peptide or lipid is covalently conjugated to the 3′-end of the passenger strand) or peptides, aptamers, antibody conjugation for tissue specificity, exomes and viral vectors are used [129,130,131,132,133]. The next challenge to the use of miRNAs in therapeutic drugs is endosomal escape during intracellular transport, which negatively affects the efficient delivery of miRNAs to the target site. The acidic environment of endosomes and the transfer of their contents to nuclease-containing lysosomes contribute to the degradation of miRNA oligonucleotides [120]. To prevent this, miRNAs must be released from the endosome into the cytoplasm, and pH-sensitive lipo- and polyplexes, photosensitive molecules and cationic nanoparticles are used for this purpose [124,134,135,136]. Once miRNAs are delivered to the cytoplasm, one of the major concerns is undesirable effects of miRNAs. Due to the fact that their main feature is targeting different pathways, they can cause undesirable silencing of other genes. Such action can contribute not only to the reduction of therapeutic efficacy of miRNAs, but may also increase toxicity [137]. A way to solve this challenge is to use low doses of different miRNAs simultaneously, which will act synergistically within the target gene [138]. Figure 3 shows downregulated and upregulated microRNAs that are used as diagnostic tools in melanoma [56].

## 8. Clinical Trials Based on MicroRNA in Cutaneous Malignant Melanoma Therapy

Doctors and scientists worldwide are constantly looking to improve the quality of diagnosis and treatment for melanoma patients. Of all the options for fighting any type of cancer, we have registered therapies, clinical trials and experimental therapies. Participation in clinical trials can provide patients with access to novel therapies many years before they become available as standard treatments. The aim of clinical trials is always an attempt to cure the disease, improve the patient’s well-being or their quality of life. Clinical trials must have specific criteria for admitting patients and be carried out in accordance with a protocol and predetermined rules. Clinical trials are used to determine if a proposed treatment is more effective and better tolerated than current treatments and should be used routinely in medicine.

Only 12 clinical trials examining miRNAs in melanoma are registered at clinictrials.gov (ClinicalTrials.gov, 2022). Up to now, four studies have been completed, two are still recruiting, and six have been withdrawn or suspended (not included in Table 1). These clinical trials differ in scope, group, miRNA target and clinical applications, but they have one thing in common: the vision of miRNA analysis in body fluids (mainly peripheral blood) and looking for the correlation of their presence and expression as a biomarker and a non-invasive and precise tool to fight melanoma. Below in Table 2, we present the results from three clinical trials that have already been completed and published.

The clinical trial of the Ruhr-University Bochum (Germany) team began with a small pilot study in 2009 and the results were published in 2011 [139]. Using immunohistochemical staining, they determined the Dicer distribution in cutaneous metastases of malignant melanoma (CMM), in patients with benign (BMN) and dysplastic melanocytic nevi (DMN). Dicer-positive staining was significantly higher in melanoma cells than in benign melanocytes. This preliminary study initiated new ideas and projects which resulted in two more clinical trials led by this team.

The research proposed by Sand et al. in 2012 [140] concerned the miRNA machinery components in three groups of skin patients—primary cutaneous malignant melanoma (PCMM), cutaneous malignant melanoma metastases (CMMM) and benign melanocytic nevi (BMN). Using a real-time PCR method they investigated mRNA expression levels of Dicer, Drosha, Exp5, DGCR8 and the RISC components PACT, argonaute-1, argonaute-2, TARBP1, TARBP2, MTDH and SND1. The results of Sand et al. showed that the miRNA machinery components argonaute-1, TARBP2 and SND1 are dysregulated in PCMM and CMMM compared to BMN. It was a very important observation, proving that argonaute-1, TARBP2 and SND1 may play a role in the process of malignant transformation. In addition, TARBP2 expression levels were significantly higher in metastases type of melanoma (CMMM) compared to primary melanoma (PCMM), and SND1 expression levels were significantly higher in CMMM compared to PCMM and BMN. Expression levels of argonaute-2, Dicer, Drosha, DGCR8, Exp5, MTDH, PACT and TARBP1 showed no significant differences within these three groups.

The same research team headed by Sand et al. in 2013 [141] published analysis results of microRNA expression sample profiles in primary cutaneous malignant melanoma (PCMM), cutaneous malignant melanoma metastases (CMMM) and benign melanocytic nevi (BMN). They proposed 19 novel dysregulated miRNA candidates in CMM patient samples, whose expression was correlated with the presence of metastases and tumor advancement. Among them, the genes hsa-miR-22, hsa-miR-130b, hsa-miR-146b-5p, hsa-miR-223, hsa-miR-301a, hsa-miR-484, hsa-miR-663, hsa-miR-720, hsa-miR-1260, hsa-miR-1274a, hsa-miR-1274b, hsa-miR-3663-3p, hsa-miR-4281 and hsa-miR-4286 were upregulated (↑), while the genes hsa-miR-24-1*, hsa-miR-26a, hsa-miR-4291, hsa-miR-4317 and hsa-miR-4324 were downregulated (↓). The results of this study confirmed the presence of dysregulated miRNAs in CMM, but to define these miRNA sequences as diagnostic tools in melanoma, or to be able to determine their role in the pathogenesis of CMM melanoma, further analyses are needed.

## 9. Future Perspective and Conclusions

Using miRNAs as a therapeutic drug is a new approach in treating many cancer diseases. Nevertheless, it can also be problematic for the host immune system, which may treat the double-stranded RNA as a pathogen and activate signaling pathways, leading to the release of pro-inflammatory cytokines. Pattern recognition receptors (PRRs), such as TLRs and RLRs, are capable of sensing RNA molecules in host cells and initiating adaptive immune responses [142]. This is an undesirable side effect, so research into miRNA-associated immune responses is ongoing. To date, it has only been shown that chemical modifications of miRNAs, such as 2′-O-Methyl, 2′- locked nucleic acid and 2′-Fluoro, cause a reduction in miRNA recognition by the immune system, but do not eliminate it completely [122]. Although radiotherapy is more often used in metastatic rather than non-metastatic melanoma, the combination of immunotherapy and radiotherapy in the treatment of melanoma has medical and therapeutic justification [143]. MicroRNA combination therapies with anti-angiogenic strategies, chemotherapy, radiotherapy, photodynamic therapy and immunotherapy were already described [144,145]. We have already been receiving many promising data on the use of miRNA, but what needs to be remembered is that before being introduced into routine use, all cancer therapies must be rigorously tested in clinical trials to ensure they are effective and safe. Many clinical trials are ongoing and are restrictively checked. A positive assessment of this stage of the research brings scientists, doctors and, most of all, patients closer to better individual therapies. Using miRNAs as a therapeutic drug is an emerging challenge for modern clinical medicine. An intriguing fact is the presence of miRNAs in many body fluids, not only in serum and plasma, but also in saliva, urine and amniotic fluid. Serum miRNAs are known to be correlated with the presence of haematological malignancies as well as solid tumors, but have been reported to also be effective in the early detection of many types of cancer, accelerating the diagnosis by conventional methods, such as anatomic imaging studies (e.g., chest X-ray, MRI, CT, PET) [146]. It has recently been shown that the follicular miRNA fraction interacts with Toll-like receptors of immune cells, stimulating the production of prometastatic inflammatory cytokines and inducing the pro-neoplastic inflammatory processes. In this scenario, circulating miRNAs may, therefore, also be receptor-activating signals. Computational analysis estimates that about 30% of protein-coding genes in humans are regulated by miRNAs and that the human genome encodes over 2500 mature miRNAs and over 200,000 mRNAs [147]. A better and more in-depth understanding of the biological and functional mechanisms of miRNAs is crucial for improving therapeutic approaches. In the future, the goal should be to develop easy tests (such as qPCR-based assays) that use a number of the most important miRNAs to determine an assessment of the risk and development of disease, including cancer. To date, numerous studies have been conducted that have made significant contributions to improving the efficacy of miRNA therapeutics, yet they have not yet reached their maximum efficiency. However, the studies that have been performed so far allow us to suggest that miRNA therapy will be crucial in the future of oncology treatment.

## Figures and Tables

**Table 1:** Plasma and tissue microRNAs as diagnostic biomarkers for cutaneous malignant melanoma (CMM).

miRNA	Plasma/Tissue	Expression Pattern	Diagnostic Utility	References
miR-1180-3p	plasma	downregulated	identification of CMM	[49]
miR-765, miR-1238, miR-1228-3p, miR-10a-5p, miR-150-5p miR-362-3p, miR-550a-3p, miR-3907, miR-500a-3p	plasma	upregulated downregulated	identification of CMM	[48]
miR-134-5p, miR-320a-3p	plasma	downregulated	identification of stage 0 and stage I/II melanoma patients	[39]
miR-424-5p, miR-548I, miR-34a-5p, miR-497-5p, miR-299-3p, miR-205-5p, miR-1269a, miR-624-3p, miR-138-5p, miR-1-5p, miR-152-3p, miR-1910-5p, miR-181b-5p, miR-3928-3p, miR-3131, miR-301a-3p, miR-1973, miR-520d-3p, miR-548a-5p, miR-548ad-3p, miR-454-3p, miR-4532, miR-1537-3p, miR-553, miR-764, miR-1302, miR-1258, miR-522-3p, miR-1264, miR-1306-5p, miR-219a-2-3p, miR-431-5p, miR-450a-5p, miR-2682-5pn, miR-337-5p, miR-27a-3p, miR-4787-3p, miR-154-5p	plasma		diagnostic biomarker of CMM, identification melanomas from lower-risk skin lesions	[40,46,55]
miR-150-5p, miR-149-3p, miR-193a-3p	plasma	upregulated downregulated	identification of CMM	[38]
miR-5694, miR-6796-3p, miR-6741-3p, miR-4664-3p, miR-4665-5p, miR-671-5p	plasma	upregulated	diagnostics of melanoma brain metastasis and distinguish from other brain metastasis and glioblastoma	[50]
miR-185, miR-1246	plasma	upregulated downregulated	identification of cutaneous melanoma metastasis	[51]
miR-211	tissue	downregulated	differentiating melanoma from the nevus group	[52]
miR-203	tissue	downregulated	identification of melanoma metastasis	[53]
miR-378	tissue	upregulated	identification of melanoma metastasis	[54]

**Table 2:** Clinical studies using microRNAs as diagnostic tools in cutaneous malignant melanoma (CMM).

Title	Status	Groups	Target	Ages	Locations	NCT Number	Reference
Immunohistochemical Expression Patterns of microRNA Processing Enzyme Dicer in Cutaneous Malignant Melanoma, Benign and Dysplastic Melanocytic Naevi	Completed	Benign Melanocytic Naevi	miRNA machinery genes—Dicer (pilot study)	>18 years	Ruhr- University Bochum, Germany	NCT00862914	[139]
		Dysplastic Melanocytic Naevi					
		Cutaneous Malignant Melanoma					
The miRNA Machinery in Melanoma, Melanoma Metastases and Benign Melanocytic Naevi	Completed	Cutaneous Melanoma	miRNA machinery genes, such as Dicer, Drosha, Exp5, DGCR8 and the RISC components PACT, argonaute-1, argonaute-2, TARBP1, TARBP2, MTDH and SND1	>1 year	Ruhr- University Bochum, Germany	NCT01444560	[140]
		Cutaneous Melanoma Metastases					
		Benign Melanocytic Nevi					
Comparative Microarray Analysis of microRNA Expression Profiles in Primary Cutaneous Malignant Melanoma, Cutaneous Malignant Melanoma Metastases and Benign Melanocytic Naevi	Completed	Cutaneous Melanoma	hsa-miR: 22, hsa-miR-130b, hsa-miR-146b-5p, hsa-miR-223, hsa-miR-301a, hsa-miR-484, hsa-miR-663, hsa-miR-720, hsa-miR-1260, hsa-miR-1274a, hsa-miR-1274b, hsa-miR-3663-3p, hsa-miR-4281, hsa-miR-4286, hsa-miR-24-1*, hsa-miR-26a, hsa-miR-4291, hsa-miR-4317, and hsa-miR-4324	>1 year	Ruhr- University Bochum, Germany	NCT01482260	[141]
		Cutaneous Melanoma Metastases					
		Benign Melanocytic Nevi					
Intralesional Influenza Vaccine for Patients With Melanoma	Recruiting (2023/12)	Metastatic Melanoma	not defined	18–90 years	Ohio State University USA	NCT04697576	-
		Cutaneous Melanoma					
		Healthy Controls					

## Data Availability

Not applicable.
